# Pseudoaneurysm of the mitral‐aortic intervalvular fibrosa with fistulous communication to the left atrium causing congestive heart failure

**DOI:** 10.1002/ccr3.4301

**Published:** 2021-06-10

**Authors:** Imad Bagh, Thomas M. Marino, Jeffrey J. Silbiger

**Affiliations:** ^1^ Icahn School of Medicine at Mount Sinai New York NY USA

**Keywords:** fistula, mitral regurgitation, mitral‐aortic intervalvular fibrosa, prosthetic valve endocarditis, pseudoaneurysm

## Abstract

Pseudoaneurysms of the mitral‐aortic intervalvular fibrosa can rupture and form fistulous communications between the left ventricle and left atrium. Pulmonary venous systolic flow reversal in such cases reflects elevated left atrial pressure due to shunting. This finding, particularly in the setting of endocarditis, is not specific for severe mitral regurgitation.

## Graphical abstract

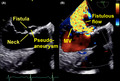

## 1 CASE PRESENTATION

**FIGURE 1:**
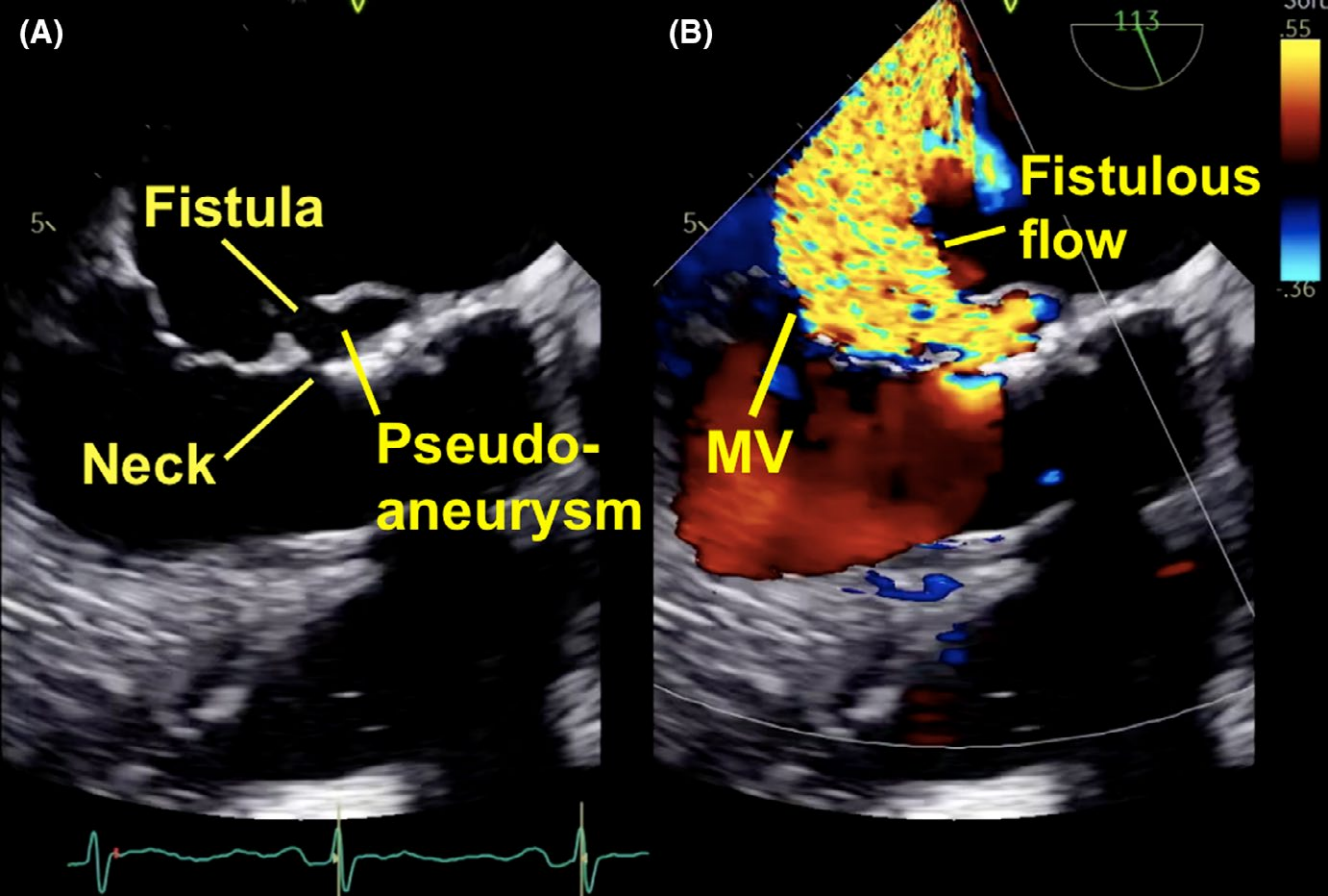
A, Mid‐esophageal transesophageal image revealing a perforated pseudoaneurysm of the mitral‐aortic intervalvular fibrosa. The perforation resulted in fistulous communication between the left ventricle and the left atrium. B, Color flow Doppler shows a large eccentric jet within the atrium coursing above the mitral valve (MV)

**FIGURE 2:**
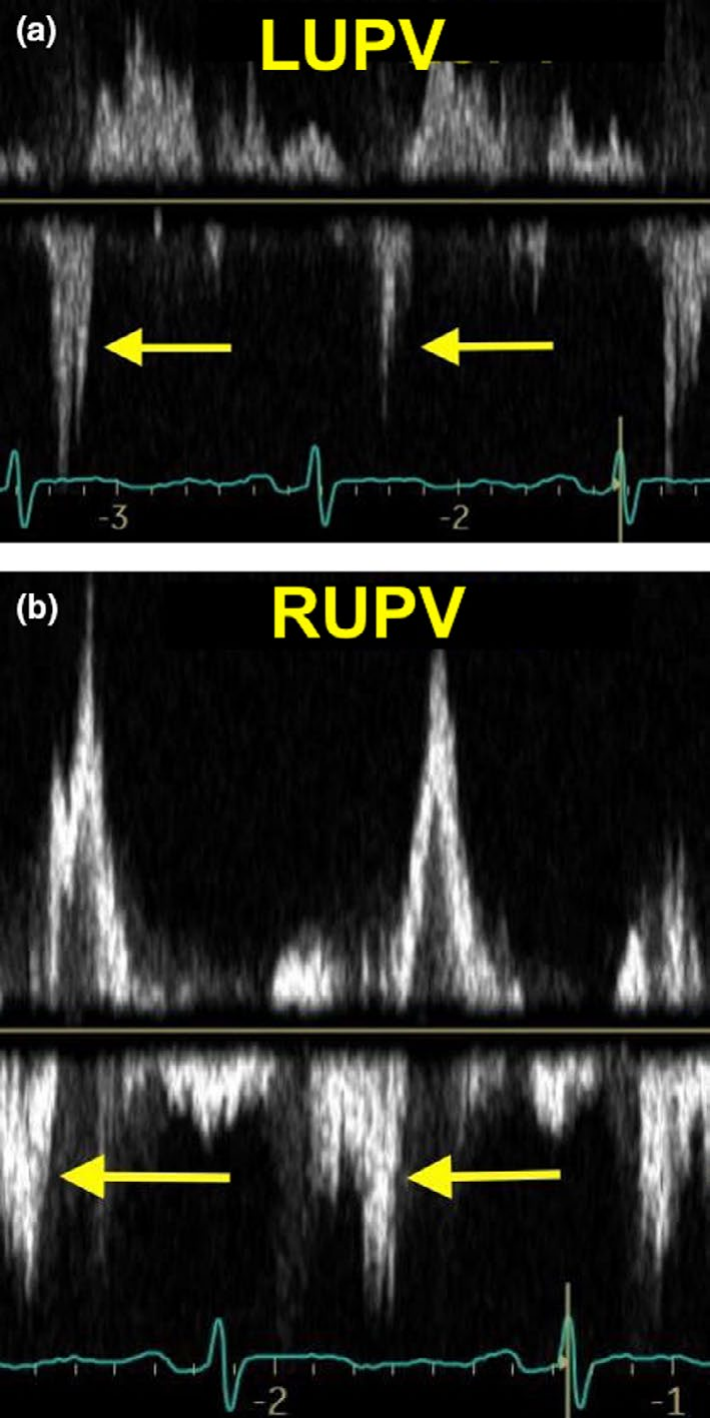
Pulsed‐wave Doppler recording revealing bilateral pulmonary venous systolic flow reversal A, Left upper pulmonary vein (LUPV). B, Right upper pulmonary vein (RUPV)

A 49‐year‐old man who underwent a Bentall procedure 3 years earlier was admitted with fever and congestive heart failure. Blood cultures grew group B S*treptococcus*. Transesophageal echocardiography revealed an unremarkable biologic prosthesis and conduit. A pseudoaneurysm was however seen in the mitral‐aortic intervalvular fibrosa (MAIF). Color Doppler interrogation revealed flow passing from the left ventricle into the left atrium (LA) through a perforation in the pseudoaneurysm (Figure 1; Movie [Link], [Link]). Massive, anteriorly directed eccentric flow was noted in the atrium which obscured the orifice of the mitral valve. Off‐axis imaging however revealed only mild mitral regurgitation (MR). Pulsed‐wave pulmonary venous Doppler demonstrated bilateral systolic flow reversal (Figure 2). The patient was treated with diuretics and antibiotics and was referred for surgery.

**2 | DISCUSSION**

Prosthetic aortic valve endocarditis is complicated by abscess formation within the MAIF in one‐third of cases.^1^ Dissection of pressurized left ventricular blood into the weakened abscess tissue can result in pseudoaneurysm formation.^2^ Pseudoaneurysms of the MAIF protrude externally between the aortic root and LA. Perforation can result in fistulous communication with either chamber.^3^, ^4^

There are several notable points in this case. First, eccentric LA flow in endocarditis does not always indicate MR. This case also demonstrates that pulmonary venous systolic flow reversal is not specific for severe MR. In our case, this reflects increased LA pressure due a shunting through a fistula. Both lesions however can cause left ventricular volume overload and congestive heart failure and represent class 1 indications for surgery.^5^

## CONFLICT OF INTEREST

None declared.

## AUTHOR CONTRIBUTIONS

All authors contributed equally in the conception and writing of this manuscript.

## Supporting information

Video S1

Supplementary Material

## Data Availability

Data sharing not applicable to this article as no datasets were generated or analyzed during the current study.
