# Supplementary material for: Pseudoaneurysm of the mitral‐aortic intervalvular fibrosa with fistulous communication to the left atrium causing congestive heart failure
Source: Clin Case Rep. 2021 Jun 10;9(6):e04301. doi: 10.1002/ccr3.4301 (PMC8190581; doi:10.1002/ccr3.4301)
Supplement: Supplementary file 2 — Supplementary Material [file CCR3-9-e04301-s001.docx]

**Movie Legend**

**Movie 1.** Blood from the left ventricle can be seen entering the pseudoaneurysm through its neck and exiting into the left atrium through a perforation.
